# Investigating the Use of Telemedicine for Digitally Mediated Delegation in Team-Based Primary Care: Mixed Methods Study

**DOI:** 10.2196/28151

**Published:** 2021-08-26

**Authors:** Michael Knop, Marius Mueller, Bjoern Niehaves

**Affiliations:** 1 Chair of Information Systems University of Siegen Siegen Germany

**Keywords:** digital health, digital health care technologies, telemedicine, user perceptions, delegation, primary care, ambulant health care, medical assistants, general practitioners, COVID-19, mixed method study, multidimensional scaling, mobile phone

## Abstract

**Background:**

Owing to the shortage of medical professionals, as well as demographic and structural challenges, new care models have emerged to find innovative solutions to counter medical undersupply. Team-based primary care using medical delegation appears to be a promising approach to address these challenges; however, it demands efficient communication structures and mechanisms to reinsure patients and caregivers receive a delegated, treatment-related task. Digital health care technologies hold the potential to render these novel processes effective and demand driven.

**Objective:**

The goal of this study is to recreate the daily work routines of general practitioners (GPs) and medical assistants (MAs) to explore promising approaches for the digital moderation of delegation processes and to deepen the understanding of subjective and perceptual factors that influence their technology assessment and use.

**Methods:**

We conducted a combination of 19 individual and group interviews with 12 GPs and 14 MAs, seeking to identify relevant technologies for delegation purposes as well as stakeholders’ perceptions of their effectiveness. Furthermore, a web-based survey was conducted asking the interviewees to order identified technologies based on their assessed applicability in multi-actor patient care. Interview data were analyzed using a three-fold inductive coding procedure. Multidimensional scaling was applied to analyze and visualize the survey data, leading to a triangulation of the results.

**Results:**

Our results suggest that digital mediation of delegation underlies complex, reciprocal processes and biases that need to be identified and analyzed to improve the development and distribution of innovative technologies and to improve our understanding of technology use in team-based primary care. Nevertheless, medical delegation enhanced by digital technologies, such as video consultations, portable electrocardiograms, or telemedical stethoscopes, can counteract current challenges in primary care because of its unique ability to ensure both personal, patient-centered care for patients and create efficient and needs-based treatment processes.

**Conclusions:**

Technology-mediated delegation appears to be a promising approach to implement innovative, case-sensitive, and cost-effective ways to treat patients within the paradigm of primary care. The relevance of such innovative approaches increases with the tremendous need for differentiated and effective care, such as during the ongoing COVID-19 pandemic. For the successful and sustainable adoption of innovative technologies, MAs represent essential team members. In their role as mediators between GPs and patients, MAs are potentially able to counteract patients’ resistance toward using innovative technology and compensate for patients’ limited access to technology and care facilities.

## Introduction

### Background

The current health care systems are facing major challenges. Several shortcomings are prevalent with regard to the availability of medical professionals and facilities, which impedes the provision of comprehensive care. In particular, rural areas are undergoing a rapid demographic change, leading to higher patient numbers and increased occurrences of age-related health issues, which results in a higher health care demand [1]. Simultaneously, the numbers of general practitioners (GPs) and specialized physicians have been decreasing in these areas [2], in part because they often find it more attractive to establish themselves in bigger cities [3]. Thus, doctors often struggle to find successors who are willing to take over their practice, which in turn leads to higher workloads for the remaining GPs who must meet the growing demand [4]. Consequently, an inequitable distribution of health care services has emerged that disadvantages rural areas that are often structurally weaker [5,6]. Nowadays, these disadvantageous trends are further complicated by the ongoing COVID-19 pandemic, which has led to governmental decisions restricting personal contacts among society as well as between patients and health care providers [7]. Therefore, a transition away from in-person treatment can be observed, evoking novel challenges that need to be addressed to maintain comprehensive access to care [8,9], such as privacy and cybersecurity concerns [10,11], the potential conduct of inaccurate examinations, or the undermining of patient-physician relationships [8].

As a reaction and countermeasure to these challenges, new care models have emerged that alter the structures and delivery processes in health care and seek to free up resources and enable GPs to cope with increasing demands and contemporary restrictions [12]. One example of a novel way of organizing comprehensive ambulant patient treatment is the deployment of medical assistants (MAs) who are entitled to an advanced set of permissions and responsibilities [13-15]. MAs are meant to take on some of the GP’s tasks, such as conducting home visits or adjusting medications (which are done in collaboration with the GP). Accordingly, these new structures and processes call for new ways of communicating, documenting, and practicing care that account for the multiple actors [16]. Although GPs need to be empowered to delegate some of their duties to the assistants to free up their own capacities and thus be able to cope with increasing demands, MAs must form ways to warrant their work, align it with medical standards and routines, and thus contribute to effective and safe treatments. Hence, new collaborative forms of care emerge, and responsibilities are partially disseminated across professionals [13]. However, this multi-actor approach also presents challenges, such as patient compliance with these novel processes. In addition, the importance of effective communication increases within team-based primary care, as MAs are not allowed to treat patients autonomously because of legal boundaries. In this regard, treatment errors and communication problems between actors can occur that call for generating digital competencies at an early stage [17] and the willingness to participate in this digital transformation.

In addition to approaches dealing with the prevalent issues in health care that involve innovative changes in personnel management and delegation, the application of digital technologies within care delivery and treatment processes has also been shown to be effective under specific conditions. Technologies such as telemedical video consultation systems for efficient patient-physician communication [18], body-worn sensory equipment that allows for patient-sided collection of vital data [19], and telemonitoring systems that render in-person contact unnecessary [20] have been applied to bridge gaps in patient treatment and in the availability of medical professionals in the workforce. In this context, the emerging internet of things brings together a variety of data collected by users and ubiquitous connected devices such as biosensors and smart meters [21,22]. Further applications of these technologies include virtual home visits [23], remote examinations [24], digital prescriptions [25], and scheduling appointments [26] and the provision of information on diseases, symptoms, and possible treatments that can be easily accessed on the web [27]. In addition, internet of things apps can be used to support older or chronically ill patients at home, thus contributing to an independent way of life [21]. In particular, with regard to the ongoing COVID-19 pandemic, telemedicine (such as video consultations and telemonitoring) has been shown to enable spatially independent treatment while ensuring quality of care and patient safety [28]. Nevertheless, research has also shown that the digitalization of primary health care processes might lead to contrary effects and requires careful consideration of underlying conditions. Research argues that digital-first approaches to general practice might lead to an increase in workload without sufficient differentiation of patients and their needs [29].

Drawing a synopsis of these two perspectives, the integration of digital technologies and new care delivery structures holds the potential to further improve health care quality and comprehensiveness, with the ultimate goal of maintaining or even improving the safety, satisfaction, and overall health of the patient. Research delivers initial insights in that regard, showing that the integration of technologies into multi-actor health care processes can yield higher allocative efficiency and organizational outcomes (eg, lower hospitalization rates) [30]. These targeted benefits are of particular interest when looking at rural areas and the prevailing circumstances, such as a lack of work force and resulting per capita demand [31]. Here, the application of digital technologies within multi-actor health care processes holds the potential to address the challenges and issues that are vital to address, such as more complex communication and delegation paths or the need for dispersed and transparent accountability [32]. Using digital technologies, GPs and MAs alike are potentially able to collect richer data on their patients based on measured vital parameters or user inputs. It can be assumed that the augmentation of innovative, multi-actor care delivery models with supportive digital technologies represents a promising and more holistic strategy, which calls for dedicated studies examining whether prevalent challenges in health care and structural disparities, such as in rural and remote areas, can be approached in a beneficial and feasible way.

### Multi-Actor Approaches in Modern Health Care

The shortage of medical professionals in Western countries has led to the development of multiple strategies to counter the difficulties related to the provision of medical services in primary care [33], especially through shifting from clinical to ambulant care [34] and through medical task delegation, that is, the transfer of medical interventions from a doctor to another medical professional [33]. Although task delegation in Western countries normally involves the transfer of medical interventions from doctors to nurses [35,36], GPs also delegate tasks to MAs in several countries [37]. Although *medical assistant* is a general term for several professionals with different medical skills and training, MAs are usually part of a GP’s staff [15]. The former role of MAs in physicians’ offices was focused on administrative tasks and the provision of isolated clinical measures under the direct supervision of physicians [38]. Currently, MAs are qualified to visit patients at home and manage different tasks, for example, monitoring a patient’s health status, taking blood samples, or supervising and adjusting the intake of prescribed medication. Although MAs are usually not permitted to diagnose patients or adjust medical treatments on their own, the role of MAs increasingly comprises the more complex task of continuously assessing and evaluating a patient’s health status to ensure optimal case management together with primary care physicians [39]. Undertaking tasks including patient education, health promotion, or monitoring the social and psychological well-being of patients, the role of MAs and primary health care nurses share specific similarities and might converge in the future [40]. Nevertheless, the differentiation and definition of the roles MAs and nurses take for primary care require further investigation and are not always clear [12,41]. Through the delegation of tasks in ambulant primary care to MAs, GPs are able to better distribute and structure their workload, resulting in a more effective and satisfactory work routine [33]. Therefore, the success of multi-actor approaches in health care depends on specific structural, organizational, and outcome-associated conditions, for example, efficient communication between team members [42] or patient satisfaction [43].

### Bridging Gaps in Primary Care Through Digital Technologies

Considering these challenges of multi-actor approaches in primary care, the digitalization of health care technologies improves the ability to catalyze team-based, multidisciplinary, and resource-sensitive processes. Considering the uniqueness of multi-actor approaches in primary care, the technologies that are relevant are those that are capable of enhancing collaboration, communication, documentation, and patient intervention. Information and communication technologies (ICTs) in particular have the potential to optimize multi-actor care processes [44], for example, by enabling remote access to patient information or documenting care through digital platforms [45]. For digitally mediated delivery of care, a wide range of telemedicine systems provide different services, from audiovisual applications for digital appointments (video consultation) [46,47] to sensory-enhanced systems for auscultation (listening to the internal sounds of the patient, usually with a stethoscope) [48]. In addition, body-worn sensors or other monitoring devices allow autonomous and continuous measurements to generate more accurate, rather than isolated, medical data [19,49]. For digitally mediated health care, it is of great interest to differentiate between the potential of different types of digital technologies and to explore factors that are crucial for their adoption and sustainable integration into existing work routines [50]. Therefore, the role of digital technologies in multi-actor approaches is bound to the evolving informational gaps that are caused by new structures and processes in primary care.

### Objectives

To date, the literature lacks studies that shed light on the potential and benefits of combining multi-actor care processes and supportive digital technologies in primary care. Hence, this study seeks to investigate the attitudes, perceptions, expectations, and needs of medical professionals located in a rural area, who play a role in multi-actor patient treatment processes. To accomplish this, our research draws upon qualitative results gathered from semistructured interviews as well as results from a web-based survey that was completed by both GPs and MAs. Both interviews and surveys were conducted in a region characterized by rural conditions and associated challenges. This mixed methods approach allows for a triangulation of findings and delivers richer insights into the target groups’ attitudes and perceptions regarding the use of digital technologies in health care, thus shedding further light on how these health care actors cope with increasing efforts provoked by the rural environment. Consequently, the objectives of this study are to investigate (1) the technologies that are suitable and effective for application in multi-actor care delivery and delegation processes in rural care and (2) the factors underlying the professionals’ use and perception of identified technologies that are already in use or exhibit future applicability.

## Methods

### Study Design

We conducted a three-step, mixed methods approach to thoroughly investigate the phenomena of interest. As part of a regional project in Rhineland-Palatinate, Germany, with 11 different primary care physicians’ offices, this study empirically explored the potential of various digital technologies for enhancing delegation processes in rural primary care. Following our initial research objectives, we (1) collected and analyzed qualitative data from 19 interviews with GPs and MAs from 11 different primary care physicians’ offices participating in the project. Through our process of analysis, we discovered that the perceived differences between technologies that were already being used by medical professionals and new innovative technologies seemed to be important factors for the hypothetical adoption or rejection of innovative technologies. Therefore, to expand our understanding of the perception of innovative digital technologies by GPs and their MAs, we (2) conducted a web-based survey within the same population and used multidimensional scaling (MDS), which will be further explained later within this section, to reveal underlying patterns of technological preferences. GPs and MAs from our sample confirmed the results of our MDS in a subsequent workshop. Finally, we achieved a richer and deeper understanding of the investigated phenomena by (3) triangulating the results of both data sets [51,52].

### Interview Study

#### Overview

As part of a regional project on the digitalization of delegation processes in German primary care, we conducted 19 qualitative interviews with GPs and MAs in 11 different rurally situated offices (we ensured to conduct at least one interview with participants from each office). All MAs that participated in this study underwent basic nonacademic clinical training for 3 years and had a supplementary qualification enabling them to undertake clinical tasks in ambulant care, comprising 190-270 hours of training. Originally, we had planned to conduct individual interviews only. Nevertheless, some offices asked us to conduct group interviews because of the high workload and time pressure. Therefore, we conducted 13 individual interviews and 6 group interviews with comparable characteristics. Table 1 provides an overview of the interviewees’ characteristics.

**Table 1:** Summary of interviewees’ characteristics.

Characteristic			Type of interview				
			Group		Individual		Total
Interviews, n (%)			6 (32)		13 (68)		19 (100)
Duration (minutes), mean (SD)			77 (23.4)		59 (16.0)		68 (20.0)
Participants, n (%)			13 (50)		13 (50)		26 (100)
Age (years), mean (SD; range)			43 (11.2; 26-59)		49 (9.2; 31-61)		46 (10.5; 26-61)
Job experience (years), mean (SD; range)			20 (9.1; 7-35)		26 (7.3; 9-37)		23 (8.6; 7-37)
**Profession, n (%)**							
	Medical assistant	8 (57)		6 (43)		14 (100)	
	General practitioner	5 (42)		7 (58)		12 (100)	
**Gender, n (%)**							
	Male	4 (57)		3 (43)		7 (100)	
	Female	9 (47)		10 (53)		19 (100)	

In addition to demographic characteristics and general questions about their profession, we asked participants about (1) their current organizational processes of delegating medical services in ambulant care, the role of (digital) technologies for these processes, and possible solutions for emerging difficulties; (2) their relationship to patients and how (digital) technologies shape or affect them; and (3) the reciprocity of self-perception and the use of (digital) technologies, that is, how participants’ own understanding of their professional role affects their attitude toward (digital) technologies. The full interview guidelines can be found in Multimedia Appendix 1. Note that not every question was asked during each interview. If a direct or indirect answer was given before the respective question was asked, it was skipped by the interviewer. In this way, we were able to reduce redundancy in the data and provide a more streamlined interviewing experience. Through these semistructured interviews, we intended to recreate the daily routines or processes of GPs and MAs regarding medical delegation and their use of technology to moderate or facilitate these routines. Subsequently, participants were asked about hypothetical scenarios involving the use of innovative digital technologies in the near or distant future, for example, video consultation or automated monitoring of medical parameters, such as blood pressure, blood coagulation, or blood glucose levels. Following previous theoretical and empirical work, we plan to outline insights into the use of innovative technologies to facilitate and assist delegation processes, as well as insights into the GPs’ and MAs’ subjective perspectives and understanding of technological effectiveness.

#### Interview Data Collection and Analysis

The regional project in Germany, which included this study, involved 11 GPs’ offices in rural areas. Following a purposeful sample [53], we included all 11 offices by conducting at least one interview (group or individual) with GPs or MAs from each office. The participating staff members from each office were contacted and interviewed by 2 different members of the research group (MK and MM). As identifying the differences between the perspectives of GPs and MAs on the use of innovative digital technologies in primary care was considered an important goal of this study, we focused on individual interviews with representatives of each profession. All interviews were conducted face to face between August and October 2019. In addition, we conducted a smaller number of focus group interviews. We did not change our aforementioned guidelines to ensure comparability throughout all interviews. Interviews were audio recorded, transcribed nonverbatim, and translated into English. As we were primarily interested in content-related insights, we did not conduct a sequential analysis and left out pauses and emotional or nonverbal sounds (such as sighs or laughter) from the transcription. The interviewees signed an informed consent form before the start of their interview.

To analyze the qualitative data, we followed a three-fold approach that was applied in a previous study in the health care domain [31]. First, 2 authors from the research group (MK and MM) independently coded each interview. On the basis of the grounded theory methodology proposed by Strauss and Corbin [54], the coding process comprises open, axial, and selective coding, which is described subsequently. This approach is particularly useful in this study because it performs well when inductively analyzing qualitative (unstructured) data with little or no previous knowledge. Hence, the highly explorative approach chosen in this study, combined with the lack of pre-existing research and insights about the phenomenon under investigation, qualified the grounded theory methodology as a fitting analysis paradigm. Following this procedure, each author started with open coding by intuitively assigning in vivo codes to the interview texts. Where possible, the first categories were formed by subsuming related open codes. Then, after going through each interview, axial codes were formed by categorizing open codes into broader schemes, thus achieving a higher level of abstraction. Next, superordinate, selective codes were formed that representatively subsumed related or redundant axial codes, which represented the theoretical core findings on a top level of abstraction. Therefore, two independent coding schemes emerged, each comprising selective, axial, and open codes. Second, in a process of comparison, the 2 authors discussed their coding schemes with regard to the research objectives and dissolved disagreements in code formulation and categorization, meaning, and code-to-text assignments. This step produced a reconciled coding scheme consisting of three overarching categories (ie, selective codes) that represent the essential findings of our qualitative analysis. In the third and final step, both authors each recoded the data by applying the novel scheme, followed by a conclusive discussion and approval of the coding procedure.

### Survey Study

#### Overview

Owing to inconsistencies in the findings from the qualitative study, which are discussed later in the *Results* section of our qualitative findings, we decided to conduct a second data collection to explore latent dimensions of subjective technology valuation by GPs and MAs. Research shows that the combination of semistructured interviews and MDS appears to be a valuable approach to gain a deeper understanding of the differences in participants’ subjective perceptions or underlying beliefs [55,56]. Therefore, we reached out to participants through a web-based survey conducted in March 2020. The survey consisted of demographics and a sorting question that asked participants to bring 10 different digital technologies into a hierarchical order following their perception of how relevant these technologies are or would be for their everyday work. The named technologies were derived inductively from our qualitative study and represented technologies that are already used for medical delegation by all offices or discussed purely for future use within our sample of 11 different offices. Therefore, these 10 technologies represent a combination of innovative digital technologies for primary medical care and technologies that were already in use by GPs and MAs. Table 2 lists the included technologies along with a short definition. Please note that the status of use in Table 2 (*technologies already in use*) reflects the time before or just at the beginning of the COVID-19 pandemic. Status of use was determined by the findings of our qualitative study and is defined by the categories *Yes* (technologies are used by all offices) or *No* (technologies are not used by any office).

To visualize and analyze nonmetric sorting data to build clusters and interpret the latent dimensions of valuation, we used MDS. Through this approach, we were able to further examine the aforementioned contradictions from our qualitative findings.

**Table 2:** Definition and summary of technologies used in the web-based survey to explore general practitioners’ and medical assistants’ latent dimensions of technology use.

Type of technology	Abbreviation used for analysis	Characteristics	Technologies already in use
Telemedical electrocardiogram (12-lead)	TelECG	Records electronic signals of a patient’s heart to assess the cardiac health status of a patient with the same quality as a stationary 12-point ECG^a^, but allows remote operation at a patient’s home through MAs^b^ and real-time data transmission to a physician	No
Electronic medical record	EMR	Collects and stores patient data electronically; helps to organize and structure medical care in clinical settings	Yes
Blood pressure monitor	BPM	Measures the blood pressure of a patient to assess information about a patient’s cardiac or general health status; can be either electronic or manual	Yes
Blood coagulation monitor	BCM	Measures the coagulation level of a patient’s blood; often used on patients taking medication to thin their blood after cardiac or neurological incidents	Yes
Telemedical stethoscope	TelSteth	Instrument to auscultate heart, lungs, or other body parts of a patient like a stethoscope, but allows remote operation at a patient’s home through MAs and real-time data transmission to a physician	No
Mobile venoscope	MobVen	A mobile instrument to detect veins and venation through transillumination; facilitates the puncture of veins	No
Smartphone	SP	Mobile phone with computer-like functions, including verbal and text-based communication, internet access, camera use, navigation, and its own operating system	Yes
Blood glucose monitor	BGM	Measures the level of glucose in patient’s capillary blood; mainly used on patients with metabolic diseases, especially diabetes	Yes
Digital appointment	DA	Audiovisual appointment (video consultation) between patient and general practitioner or patient and MA to digitally assess a patient’s health status	No
Infrared thermometer	InfTherm	Measures the body temperature of a patient; indicates inflammatory processes in a patient’s body, for example, infections	Yes

^a^ECG: electrocardiogram.

^b^MA: medical assistant.

#### Survey Data Collection and Analysis

As this study aimed to determine the underlying dimensions of perception participants had to judge the value of a specific technology for their work, we contacted participants from our qualitative study and asked them to participate in our additional web-based survey. Therefore, we ensured a purposeful sampling approach within the same population of participants to draw conclusions from both data sets appropriately. Therefore, 2 members of the research group (MK and MM) contacted participants from the qualitative study who were then asked to participate in the survey study. The participants started the survey by providing their consent. From the initial 26 GPs and MAs participating in our qualitative interviews, 14 (54%) responded to our web-based survey, from which three data sets had to be removed because of incomplete data. The demographics of the remaining 11 participants are summarized in Table 3.

MDS originates from psychological research, which has been used as an explorative approach for determining latent dimensions of nonmetric or metric data. It has been applied in different contexts regarding technology use, such as to differentiate between types of e-marketplaces [57] or to address technology-related phenomena in organizational research [58,59] and, more recently, to explore latent dimensions for context-specific technology use, such as cyberdeviance [60]. Owing to its applicability to behavioral phenomena and its ability to operate with small sample sizes [61], MDS was selected as an applicable statistical method to enrich our insights from the qualitative interviews.

**Table 3:** Demographic information about participants from the web survey.

Characteristics		Values
**Gender, n (%)**		
	Male	4 (36)
	Female	7 (64)
**Profession, n (%)**		
	Medical assistant	5 (45)
	General practitioner	6 (55)
Age (years), mean (SD; range)		45 (12.3; 27-62)
Job experience (years), mean (SD; range)		21(9.1; 9-34)
Patient size of physician’s office (number of patients treated per year), mean (SD; range)		4940 (1876.3; 1000-7600)

In an MDS configuration, objects are represented as points in a multidimensional space (usually 2D or 3D). The distances between the points correspond to the empirical dissimilarity of the objects [62]. Therefore, MDS transfers dissimilarity data into a geometrical configuration with m dimensions (m ∈ N). Thereby, dissimilarity data can be represented by pairwise ratings of objects, intercorrelations, or hierarchically sorted preferences [62]. Through a specific mathematical algorithm, dissimilarity data are then used to generate an optimized one- or multidimensional configuration representing the starting point for theoretical reasoning. For our second data set consisting of technologies that participants sorted by their subjective perception of relevance for their daily work routines, we used a special type of MDS called multidimensional unfolding (MDU) [62,63]. MDU creates an optimized geometrical representation of participants, together with their subjective perceptions of technologies, enabling a visual interpretation of the latent dimensions of these perceptions. We used the R Foundation’s statistical software R [64] with the package smacof [65] for statistical analysis. The geometrical configuration was calculated using the Euclidean distance function to define the distances between included objects. Euclidean distances were calculated using the formula for Minkowski distances *d_ij_* (X) while setting the distance’s order p=2.




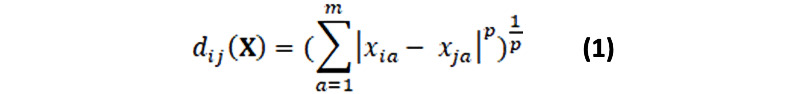




Eucledean distances allow an intuitive interpretation of geometrical configurations, as they represent our natural understanding of distances [63]. For instance, the Euclidean distance between two points in a 2D space is determined by a direct line between these points. In an MDU configuration, small distances represent high similarities between objects and vice versa. The dimensional suitability of our statistical solution was evaluated by comparing the *stress−1* values for different numbers of dimensions [62,66]. *Stress−1* values yield a normed indicator for the differences between the distances within the actual configuration *d_ij_* (X) and a function *f* (*p_ij_*) with distances 
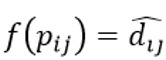
, called disparities, representing the ideal model for the empirical data [63].




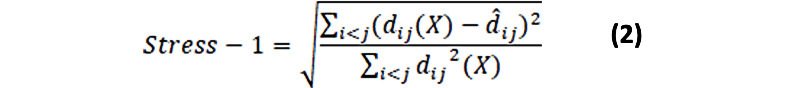




Compared with error functions from other statistical methods, such as regression analysis, a minimization of the *stress−1* value (convergence toward 0) is desirable. Furthermore, we evaluated the number of iterations required to calculate the configuration.

## Results

### Results From Semistructured Interviews (Interview Study)

#### Overview

From our qualitative interviews with GPs and MAs, three main categories emerged for discussing the potentials of digital technologies in facilitating delegation processes: direct patient treatment, documentation and communication of treatment, and contrast of personal interaction and telemedicine. The main categories represent the highest level of content-related interpretation, following our approach for qualitative data analysis.

#### Direct Patient Treatment

Our first category reflects participants’ perspectives on the important role of digital technologies for medical diagnosis and treatment through delegation. While recreating their daily work routines, GPs and MAs primarily described technologies that they already use to take direct measurements of medical parameters (mainly blood glucose levels, blood pressure, and blood coagulation levels) of patients in ambulant medical care. When considering the ongoing digitalization of the measurement of vital parameters (eg, continuous automated monitoring of blood glucose levels or the automated data transfer of blood pressure measures to a patient’s GP), participants evaluated present and future scenarios based on accuracy and instantaneousness of measurement, as well as the usability of technologies. For the measurement of blood pressure, GPs and MAs primarily use a combination of blood pressure cuff and stethoscope, both manual technologies. As digital versions of these measurement tools are easier to use but do not provide the same accuracy, GPs and MAs continue to use manual technology:

Sometimes, a patient has horrible blood pressure measurements [while using a digital blood pressure monitor], so you go to visit him. Then you measure it [blood pressure of the patient] yourself and tell the patient to put a new battery in it or something like that, so maybe it will take a better measurement next time.

Interview 16, GP

Concerning the measurement of blood coagulation levels, both GPs and MAs mentioned the importance of technological innovation for the feasibility of delegation processes in ambulant care and time-efficient treatment processes. In the past, GPs or MAs had to take blood samples through venous punctuation and send them to a medical laboratory to determine a patient’s coagulation level. Digital versions of blood coagulation monitors can now instantly and accurately measure the parameters using capillary blood. Therefore, digital coagulation monitors are currently used almost exclusively for ambulant care:

In the past, . . . GPs had to visit the patients and had to take blood samples for coagulation, even before consultation hours. In the afternoon they had to see the patients again . . . it was very complicated, and now it’s easy, isn’t it?

Interview 15, MA

Similar to blood coagulation levels, the blood glucose levels of patients are also usually measured with digital equipment, as only capillary blood is needed for an instant analysis and accuracy does not vary much in comparison to venous sampling. GPs mentioned that MAs were competent enough to assess whether a patient’s blood pressure, blood glucose level, or blood coagulation level necessitated notifying the GP about the patient’s health status. While discussing potential digital innovations for these kinds of technologies, such as automated data transfer or push notifications in case of an unusual deviation in a patient’s vital parameters, GPs preferred receiving the subjective interpretation from an MA through a direct phone call, as GPs were likely to ask additional questions about a patient in a potential emergency case:

They [MAs] give me a call when something’s wrong. . . . and they’re very quick—quicker than some typed message that I maybe wouldn’t hear. In this situation, automated data transfer isn’t of any use. When a patient has a blood glucose level of 60 [hypoglycemia], they know what to do; they know it’s too low and they have to do something.

Interview 14, GP

Aside from already familiar technologies, participants discussed in detail the potential of two innovative digital technologies for ambulant medical care: mobile telemedical electrocardiograms (ECGs) and telemedical stethoscopes. In contrast to monitors for blood glucose levels, blood pressure, and blood coagulation levels, the interpretation of ECGs or auscultation sounds is highly complex and is usually not delegated to MAs. However, MAs are competent in recording patient data for the GP:

I can’t evaluate it [ECG], that’s the doctor’s business . . . I can put it on a patient . . . and then I bring it to the office. There it’s evaluated and the doctor decides what to do with it.

Interview 15, MA

Therefore, a mobile version of an ECG that is able to transfer an ambulant patient’s data in real time to the GP’s office (hereafter called a telemedical ECG) was discussed in the interviews as an innovative technology that could be used in daily work. Mostly, GPs and MAs believed that this technology would be helpful in recording and analyzing a patient’s ECG data remotely. Especially when an ambulant patient’s medical issues occur spontaneously, MAs liked the idea because they could reassure themselves and the patient through a direct evaluation of the ECG data:

To transfer ECG data, that would be great. I’d like that a lot, I could imagine, to somewhat delegate medical problems . . .

Interview 2, GP

Nevertheless, accuracy remained an important factor for GPs and MAs in deciding to actually use a telemedical ECG, as they emphasized the necessity of a telemedical ECG to generate a quality of medical data that are comparable with state-of-the-art stationary ECGs (12-lead). Although participants found the idea of a telemedical ECG highly interesting and relevant, the GPs were especially pessimistic about the cost-effectiveness. Owing to the high purchase prices and the lack of reimbursement by social health insurance, numerous GPs formulated resistance to actually purchasing a telemedical ECG:

If I had one [telemedical ECG], that would be really helpful, but you have to consider that I couldn’t even charge something for the use of it. . . . So alas, it’s a cost-benefit analysis once more.

Interview 18, GP

Similarly, the opportunity to auscultate ambulant patients from a distance by instructing qualified MAs on how and where to put a digital stethoscope on the body of a patient seemed to be of high interest for GPs. By enabling the collection of medical data and real-time transfer to a GP’s office, telemedical stethoscopes were seen by GPs as a likely way to save time:

Well, it surely would make my work easier, if I don’t need to visit every older patient with a cold anymore, if I could just auscultate them remotely.

Interview 19, GP

GPs and MAs mentioned that a telemedical stethoscope could be practical because of its variety of uses. Telemedical ECGs are used solely for medical issues involving functional cardiac abnormalities. Telemedical stethoscopes’ indications include cardiac, pulmonic, and unspecific medical problems.

#### Documentation and Communication of Treatment

Our second category contains the participants’ discussions of the potentials arising from digital documentation and communication. GPs and MAs emphasized the importance of mobility and ubiquity of information, but also mentioned hindrances for the sustainable use of digital technologies. While discussing the digitalization of communication about patients and their treatment processes, GPs and MAs pointed out the role of direct contact between GP and MAs in daily work routines. As MAs might unexpectedly encounter a patient with severe medical issues, all of the included GPs’ offices provided an emergency call system. Thus, MAs can talk to a GP at any time if they think it is necessary. In addition, participants talked about the use of private messaging systems for cases in which communication was not urgent. As one of the most recent widely discussed innovations in primary care, digital telemedicine systems that enable audiovisual communication (video consultation), combined with the remote transfer of medical data in real time (eg, telemedical stethoscopes) were perceived as helpful for delegation processes by GPs and MAs. Three different benefits were primarily associated with telemedicine systems: replacing a GP’s home visit through digitally mediated delegation, improving (ad hoc) diagnostics in ambulant medical care, and reassuring MAs in ambulant medical care. Although GPs were skeptical about fully replacing direct bilateral contact with a patient through telemedicine, telemedicine-mediated interactions involving MAs were considered helpful in some situations:

If I could actually have a look at the patient, like in a video conference, and our medical assistant asks some additional questions and I got some relevant parameters, I could really imagine saying, “Well, the patient’s all right; he’s stabile, so there’s no need for me to visit him.”

Interview 2, GP

In addition, GPs mentioned that they would not rely on a patient’s ability to use a telemedicine system for diagnostic purposes on their own; for example, as the use of telemedical ECGs might be complex, and potential misuse might lead to misleading information. Therefore, the medical competence of MAs was discussed as an important factor for using digital technologies to potentially improve ambulant medical care and overcome a patient’s lack of competence in adequately using such technology. Telemedicine systems were considered by MAs to be useful as an innovative channel of communication that can help to better determine the potential diagnosis of a patient after they initially assess the patient’s health status:

If I could send some data directly to the doctor, that would make things easier. So that telemedicine, when I put on an ECG, the doctor might tell me from his desk, if it’s alright or not, if we have to call an ambulance or if he needs to visit the patient by himself.

Interview 10, MA

Through telemedicine systems, MAs saw the possibility of contacting a GP regarding a medical situation that they were not fully able to assess. Closely related to the improvement of the quality of a diagnosis in delegation processes, telemedicine seemed to provide a feeling of security and reliability:

When facing a critical situation, I think it [video consultation] could help me to feel safe. Because I’m usually alone on-site . . . I think I would be more confident when I think the patient’s not looking good, I better turn it on and the doctor sees what I see.

Interview 6, MA

Although telemedicine systems, therefore, appear to be helpful for direct communication in ambulant medical care, health records are used to collect and sort patient information and data over a longer period of time. Participants discussed the use of mobile electronic health records (EHRs), and MAs especially pointed out the importance of having access to relevant patient health records in ambulant medical care. As direct contact with a GP is usually reserved for critical situations, MAs discussed their need for a medium through which to collect relevant information about a patient’s health status. Although all participating offices installed some type of EHR in their systems, remote access to patient information seemed to be difficult, as mobile versions of EHR were not installed on suitable devices or their actual use was impractical. Nevertheless, participants emphasized the potential benefits of easy-to-use mobile documentation that could make manual documentation obsolete:

. . . and then I write it down, write it all down and sometimes I can’t figure out my handwriting afterwards and therefore it [mobile EHR] would be useful. So you can write it down directly and be connected to a patient’s entire medical history.

Interview 18, MA

Although some participants mentioned that manual documentation was carried out very quickly while making home visits, most participants reported the necessity for double documentation later:

. . . here [doctor’s office], you’re sitting for hours to write down everything you did on home visits. It takes a lot of time. So, if you could just make your documentation while you’re still on home visits, that would save a lot of time.

Interview 9, MA

Furthermore, GPs remarked on the lack of interoperability, not only between mobile software apps and applications in their office but also between their own documentation software and the software used by hospitals, nursing homes, or other GPs. Especially when patients move from clinical to ambulatory care, GPs pointed out that transferring the patient’s clinical data or updating medication takes a lot of time because the inability to transmit records electronically means that it must be done manually:

When a patient comes to me with his clinical reports, that’s a catastrophe. You get hand-written reports, . . . you have to transfer into the EHR. Also, it doesn’t work with ambulant nursing care, it doesn’t work with nursing homes.

Interview 8, GP

Communication and documentation technologies seem to be essential factors for a dynamic and uninterrupted workflow, from the perspectives of GPs and MAs. Although telemedicine systems enabling audiovisual communication between the GP, MA, and patient or real-time remote transfer of medical data (ECG and auscultation sounds) are recognized as helpful digital innovations, the digital technologies that are already in use (EHR) do not seem to have reached their full potential because of a lack of interoperability and user-friendly mobile apps.

#### Contrast of Personal Interaction and Telemedicine

In our third category, we subsumed participants’ discussion of the risks and limitations of digital technologies concerning team-based primary care and the interaction between them and patients. Aside from considerations about the usefulness of digital technologies regarding direct patient interventions or superordinate processes of medical care, participants perceived the use of technology as contextual, that is, directly or indirectly embedded in a specific social interaction. Although the positive aspects of telemedicine system use were discussed, most participants were skeptical about replacing direct physical interaction with digitally mediated interaction:

It makes a difference . . . It’s not the same. I think the gold standard is a direct encounter, to be in the same room; that’s just different to [audio-visual telemedicine].

Interview 4, GP

Participants further differentiated the limitations of telemedicine use into those resulting from a perceived restriction of relevant patient data (eg, skin conditions, walking behavior, or general appearance) and those resulting from the absence of bodily contact itself. In particular, GPs emphasized their need to use multiple sensory inputs to correctly diagnose a patient:

Well, I don’t know how he [the patient] smells, I can’t have a look at his skin. Is he sweating? Is his skin cold not supplied with enough blood? I want to examine him [physically]. And these are things that are most relevant for the diagnosis you find, in the end. For me, it plays an important role.

Interview 7, GP

Some MAs argued that not only is the ability to evaluate the physical conditions of a patient reduced by digitalized home visits but also the ability to evaluate environmental factors, such as the general condition of a patient’s home or the presence of objects that could potentially increase the fall risk in older patients:

Also, it’s about fall risk; I have an eye on that. There might be a new carpet, causing a risk or cables lying in a patient’s way. I talk to the patient about these things, or his relatives.

Interview 8, MA

Aside from more objective limitations, GPs and MAs discussed the meaning of bodily contact with a patient as a part of the social interaction itself. Participants mentioned the importance of direct contact with patients, not just to medically treat them correctly and comprehensively but also to form a relationship with them. Participants were not able to completely explain what underlying assumptions lead them to the impression that a direct, nondigitally mediated interaction is preferable to the use of telemedicine for the purpose of social interaction. Nevertheless, they emphasized the advantage of direct contact with the patient to build a relationship in which trust can be created and patients feel safe to talk about personal problems:

The bodily presence. The contact . . ., especially for older patients. My job is especially about old people. They need it. Or maybe some joking or something like that. You won’t do that when you’re on the computer. When you’re sitting directly with each other, then some things are discussed. And that’s missing while using telemedicine. If everything was to be digital, something would be missing.

Interview 10, MA

In contrast, participants also considered the social effects that the use of technology itself has on patients, for example, the feeling of security and control when blood pressure or blood coagulation are being monitored. In particular, GPs compared the positive effect of technological use on patients with the placebo effect known from medication:

The patient has a good feeling, then. Technology is always great. Something beeps, some additional measurement for some specific symptoms. Technology has something like a placebo effect.

Interview 5, GP

In addition, participants assumed that technology adoption by patients was highly affected by the attitude of the attending GP or MA. Participants reasoned that the technological adoption of patients might depend on the formulated medical necessity and the explanation given by the GP or MA:

But they don’t really ask many questions about it [long-term blood pressure monitor or ECG]. We tell them how it is done, how it works. And then it’s all right, so it’s very uncommon that someone asks questions about it. They rely on what we said about it.

Interview 10, MA

In summary, GPs and MAs were aware of the factors that influence the relationship between them, the patient, and the use of technology. Participants reflected on the potential effects of innovative technologies on social interaction and discussed the limitations from their point of view. Interestingly, our three main categories, therefore, represent three different dimensions of technological use in ambulant medical delegation: (1) an interventional dimension involving direct patient contact, defining the action of care; (2) a superordinate dimension of communicating and documenting care; and (3) a reflective social dimension in which participants discussed contextual and relational factors of technology use.

In the process of analyzing and summarizing the qualitative findings from the focus group and individual interviews, contradictions were identified between the interviewees’ perceptions of innovative technologies and technologies that were already in use. Several times during the interviews, participants seemed to reject innovative technologies (eg, telemedicine systems used to contact patients from a distance) while using specific arguments (eg, risk of private data misuse) that they did not apply to technologies that were already in use (eg, smartphone use to communicate patient data). As the underlying mechanisms of subjective technology valuation must be considered an important factor for technology use [67], in an effort to answer our initial research questions, we decided to conduct our survey study to explore latent dimensions of subjective technology valuation by GPs and MAs.

### Results From MDU (Survey Study)

By applying MDU, we explored the underlying dimensions of the GPs’ and MAs’ perceptions of the usefulness of relevant technologies in facilitating the process of delegation in medical ambulant care. Qualitative results suggest the various categories and factors that GPs and MAs use to evaluate different types of technologies; the subjective motivation and individual perception of technologies help to fully understand and explain the behavioral intention and the actual use of technology by medical professionals. By merging the results from both the qualitative and survey studies, we intended to contribute to a comprehensive understanding of the role of digital technologies in primary care delegation processes.

To determine the number of dimensions that are appropriate for interpretation, we analyzed the *stress−1* values as well as the number of iterations for different MDU configurations (Table 4). Although values for *stress−1* decreased with greater numbers of dimensions, iterations for dimension m=4 reached its maximum of 10,000 and were excluded after visualization from potential solutions because of its obvious triviality. For all calculated configurations, we followed the suggestions of de Leeuw et al [65] and Busing et al [68] to avoid degenerate solutions.

**Figure 1:**
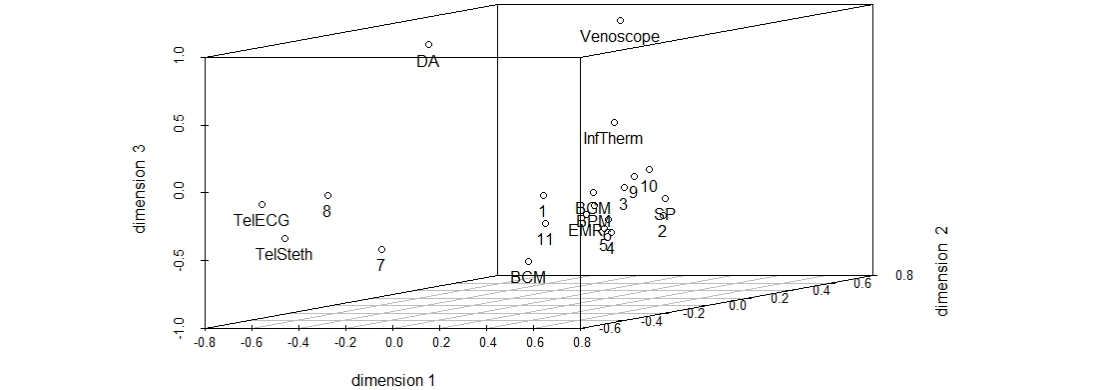
3D configuration of technologies and participants to visualize the perceived technological relevance for daily work routines (numbers represent participants, words represent different technologies). BCM: blood coagulation monitor; BGM: blood glucose monitor; BPM: blood pressure monitor; DA: digital appointment; EHR: electronic health records; InfTherm: infrared thermometer; SP: smartphone; TelECG: telemedical electrocardiogram; TelSteth: telemedical stethoscope.

As it showed the lowest *stress−1* value, we selected the configuration with m=3 for further analysis and interpretation. As a *stress−1* value of 0.18 represents an approximately correct solution, we tested the reliability of the configuration in relation to the data by calculating a random solution for m=3 (number of iterations=1) and compared it with the initial configuration [63]. The random solution had a *stress−1* value of 0.38. As the *stress−1* value of our initial 3D configuration appears to be much smaller [63], we considered our result to be satisfactory. Figure 1 shows a 3D depiction of the resulting configuration.

**Figure 2:**
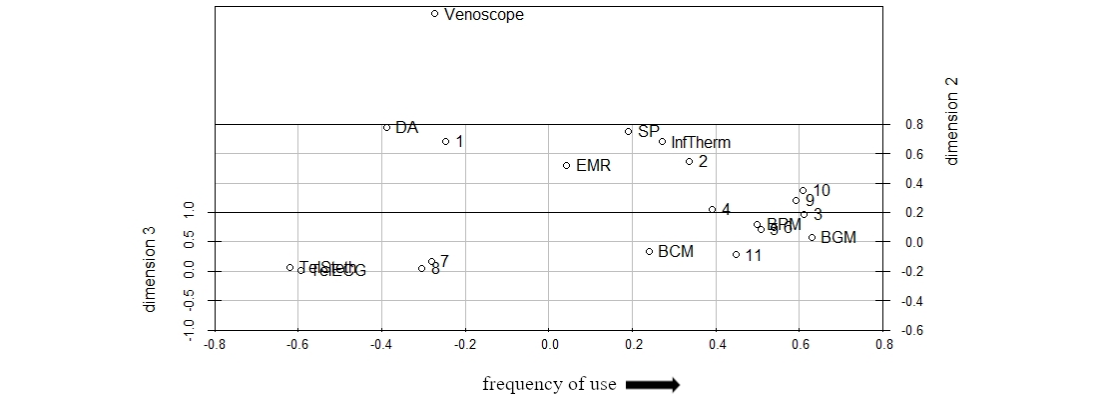
3D configuration with fixed first dimension (numbers represent participants, words represent different technologies). BCM: blood coagulation monitor; BGM: blood glucose monitor; BPM: blood pressure monitor; DA: digital appointment; EHR: electronic health records; InfTherm: infrared thermometer; SP: smartphone; TelECG: telemedical electrocardiogram; TelSteth: telemedical stethoscope.

**Figure 3:**
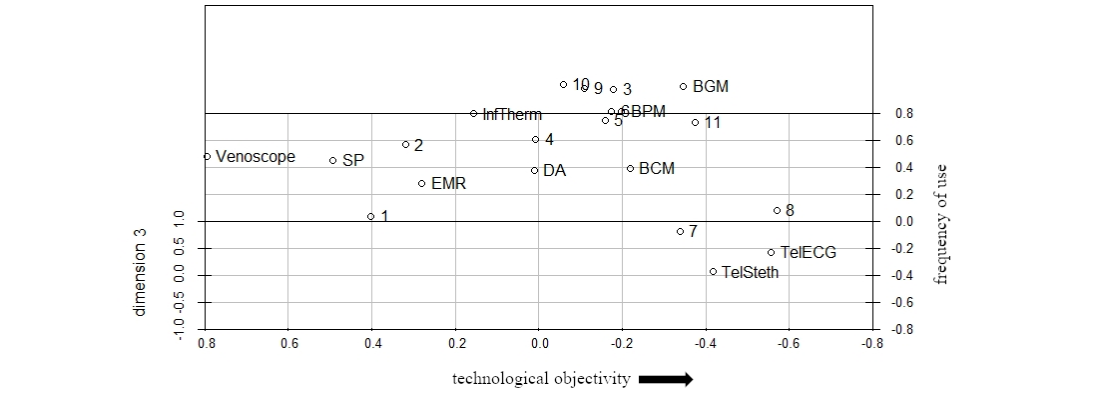
3D configuration with fixed second dimension (numbers represent participants, words represent different technologies). BCM: blood coagulation monitor; BGM: blood glucose monitor; BPM: blood pressure monitor; DA: digital appointment; EHR: electronic health records; InfTherm: infrared thermometer; SP: smartphone; TelECG: telemedical electrocardiogram; TelSteth: telemedical stethoscope.

**Figure 4:**
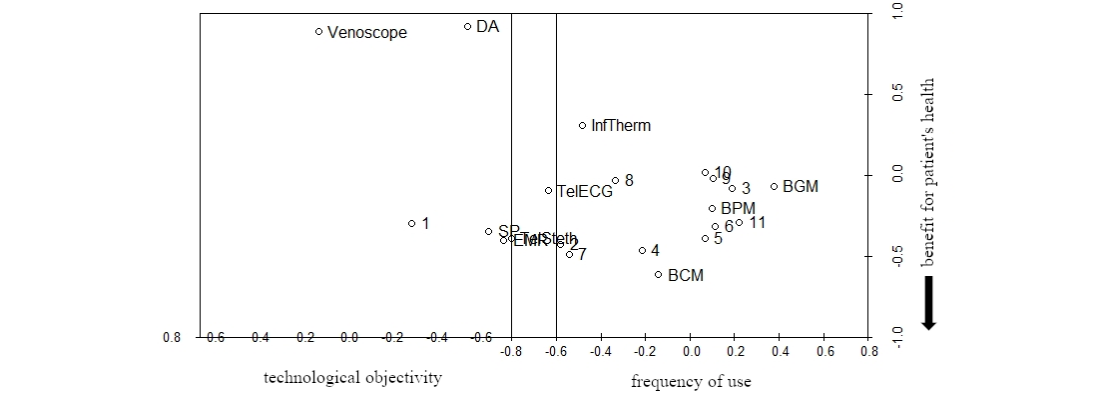
3D configuration with fixed third dimension (numbers represent participants, words represent different technologies). BCM: blood coagulation monitor; BGM: blood glucose monitor; BPM: blood pressure monitor; DA: digital appointment; EHR: electronic health records; InfTherm: infrared thermometer; SP: smartphone; TelECG: telemedical electrocardiogram; TelSteth: telemedical stethoscope.

To provide a possible interpretation of our results, we changed the angles of our selected configuration and fixed one dimension after another (Figures 2-4) using the R package scatterplot3d [69]. Demographic control variables (eg, age or gender) did not lead to a sufficient explanation of the following dimensions and were therefore not discussed as potential criteria for interpretation.

**Table 4:** Summary of different configuration characteristics.

Dimension	Number of iterations	*stress−1* value
1	12	0.41
2	62	0.24
3	113	0.18
4	10,000	0

In Figure 2, we propose the underlying dimension *hypothetical* and *actual frequency of use*: innovative digital technologies that were not yet in use at the time of data collection, such as telemedical stethoscopes and ECGs or video consultation, are sorted to the left of the dimension *frequency of use*, whereas frequently used technologies, such as blood pressure or blood glucose monitors, are sorted to the right. Notably, the venoscope appears to be near innovative digital technologies that were not yet in used at the time of data collection, as GPs and MAs of our sample usually do not use it in ambulant medical care. Therefore, the perceived relevance of technological use seems to be strongly associated with daily work routines and the possible incorporation of specific technologies. Although most of the participants are sorted to the right, meaning that they tend to find the technologies that they already use to be relevant, participants 1, 7, and 8 are sorted near innovative digital technologies. Therefore, most participants take into account their current behavioral routines when evaluating the relevance of a specific technology, whereas a minority of participants forecast a frequent use of innovative technologies whose characteristics and benefits they only assume but do not have experience with.

Considering Figure 3, we propose *technological objectivity* as an explanation for the second dimension. Except for the infrared thermometer, technologies sorted on the right, such as telemedical ECG or blood coagulation monitor, measure a patient’s more objective medical parameters, whereas technologies to the left, such as smartphones or EHR, are not used to directly assess a specific medical parameter, but rather to facilitate the communication and documentation of care. Participants are distributed more equally among the second dimension than among the first dimension, indicating that the perceived relevance associated with *technological objectivity* is a more subjective factor and that participants have a relatively ambivalent perspective on *technological objectivity*. Furthermore, an accumulation of most participants in the middle could indicate that *technological objectivity* might not directly increase the subjective relevance of technology use but that it depends on the specific context in which the technology is used. Overall, this dimension might represent the individual perspective of participants regarding the relevance of objective medical data.

Finally, we propose *benefit for a patient’s health status* as an explanation for our third dimension. Considering the technologies that appear at the bottom of Figure 4, for example, blood coagulation monitoring or telemedical stethoscope, participants might connect the characteristics of these technologies with the benefits they could have for a patient, especially in critical medical situations, as blood coagulation levels or auscultation sounds are strong indicators of a patient’s health. Seemingly distorting our interpretation of the third dimension, the arrangement of smartphones and EHRs toward the bottom of our configuration might be explained by the smartphone’s ability to connect the user to other medical professionals, especially in a medical emergency. Meanwhile, an EHR could be essential for GPs or MAs in interpreting or classifying specific symptoms of a patient with regard to their medical history and, therefore, essential in arriving at an appropriate diagnosis, especially in critical situations. Participants accumulate in the middle of the third dimension, indicating that the perceived benefit from a patient’s perspective can be seen relatively within a specific range of interpretation. Therefore, the third dimension of our configuration partially represents the professional identity of GPs and MAs and their motivation to use (digital) technologies arriving at a specific treatment objective to create a benefit for patients.

**Figure 5:**
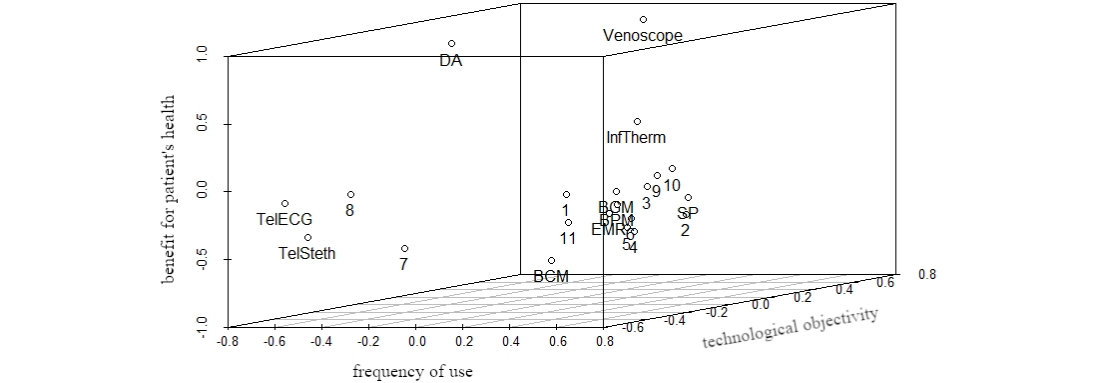
3D configuration with interpreted dimensions (numbers represent participants, words represent different technologies). BCM: blood coagulation monitor; BGM: blood glucose monitor; BPM: blood pressure monitor; DA: digital appointment; EHR: electronic health records; InfTherm: infrared thermometer; SP: smartphone; TelECG: telemedical electrocardiogram; TelSteth: telemedical stethoscope.

In accordance with the initial objective of our survey study, our results suggest three latent dimensions of subjective technology valuation by GPs and MAs. First, participants appear to assess the (potential) value of a technology by its extent of practical implementation, that is, the *hypothetical* and *actual frequency of use.* Second, the purpose of a specific technology appears to be another major reference for participants to decide whether a specific technology is relevant to their daily work routines or not. Here, participants differentiated between technologies primarily assessing objective medical parameters and those primarily used to communicate or document, that is, *technological objectivity.* Finally, the subjective valuation of technology appears to be dependent from the participants’ impression of whether it creates values from the perspective of patients and their treatment, that is, *benefit for a patient’s health status*. Figure 5 represents the final 3D configuration, including our interpretations.

To ensure that the dimensions of participants’ perceptions are valid, we discussed and reflected our findings from MDU with 17 physicians and MAs from our sample, as recommended by the literature [56,57], during a project-related workshop in November 2020. Participants confirmed our results from their professional perspective by emphasizing that our derived dimensions are plausible factors for their perception of technological relevance and effectiveness.

## Discussion

### Overview

Reflecting the results from both the semistructured interviews and the survey study, we discussed the role of medical delegation for future primary care and the preconditions for a sustainable and effective implementation. Thereafter, we reasoned about specific aspects that are worth considering in the process of digitalizing primary care through mediated delegation and are needed to reflect possible (unintended) changes regarding the interaction between patients and medical professionals, that is, the subjectivity of medical data and the key role of MAs. Finally, we discussed possible challenges in the process of digitalizing primary care through mediated delegation and implications for theory and practice.

### Mediated Delegation

As it is an essential prerequisite for multi-actor care processes, our data show that GPs attribute high degrees of competence and expertise to MAs when it comes to assessing a patient’s health status and interpreting symptoms and parameters. Hence, MAs can pre-evaluate patient data and communicate it to the GP. In this process, both sides benefit from the integration of telemedical tools, such as digital measurement equipment and sensors, as the objective data they produce better inform the assessment of MAs. As a result, these tools expand the scope of what a GP can delegate and what an MA can actually do on site with the patient. As proposed by the diffusion of innovations model [70], the adoption of a technology also depends on the type of decision-making process. In particular, collective evaluations and decisions as they are made in multi-actor networks can facilitate higher adoption rates once required attributes, such as compatibility, are universally perceived. Here, compatibility plays a major role as the MA’s competencies and working styles as well as the technology used need to fit the delegated task and requirements—both on an organizational and medical level—that come with it. Accordingly, the symbiosis of an MA gathering subjective and valuable impressions of the patient and technologies collecting objective data enables a more holistic image of the patient to be given to the GP, as our findings indicate that data of both subjective and objective nature are essential to arrive at a proper diagnosis and successful treatment protocol.

### Demands and Requirements on Digital Technologies for Telemedicine

Another important factor that underlies the GPs’ and MAs’ perceptions of the use of technologies in direct patient care is the accuracy and instantaneousness of the applied sensors, the devices, and the resulting data. The participants stressed that the accuracy and quality of data is a major prerequisite for using digital technologies. For the evaluation of practicability and usefulness, GPs and MAs seem to consider the quality of medical data generated from a specific technology. Although usability might be relevant for technology use in general, participants rejected easier-to-use digital technologies because of their perceived lower precision. Furthermore, the objectiveness of technology is associated with participants’ subjective perceptions of technological relevance, implying that the development of digital technologies for measuring medical parameters should address not only innovative ways to measure but should also ensure at least a constant quality of data compared with already elaborated technologies and procedures. Moreover, the consideration of legal frameworks or boundaries at an early stage in the development process might increase the adoption of innovative digital technologies because of the GPs’ desire for cost-effectiveness, as the reimbursement of technology-mediated treatment by (social) health insurance companies seems to be an important factor for medical professionals when assessing a technology’s usefulness [71]. In addition, financial support for the implementation of innovative technologies by public institutions might be a vital measure to counter the increasing workload and complexity of primary care. These findings align with current research on the success of eHealth interventions, identifying limited financial resources as a major obstacle [72].

### Subjectivity of Medical Data

When it comes to the interpretation of medical data, our results suggest that GPs and MAs consider several subjective and environmental factors to be highly relevant, such as the state of a patient’s home, medical history, or complex sensory impressions. Although common telemedical solutions such as video consultation systems enable practitioners to derive an initial picture of the patient based on what the camera captures and what the patient verbalizes, such technologies are still perceived as limited [73], particularly by hindering the continuance of care [74]. With digital technologies enabling the remote evaluation of a patient’s health, the importance of combining or merging different technological characteristics becomes apparent, that is, the development of multichannel systems to provide not only quantified data but also a comprehensive image of a patient as well as platforms or opportunities for social interaction between the GP, MA, and patient. Consulting the diffusion of innovation model [70], it becomes apparent that a high level of complexity that comes with introducing a novel technology into multi-actor processes, paired with a lack of observability due to subjective assessments, highly individual routines, and tacit knowledge of involved actors, can further hamper the comprehensive adoption of telemedicine in multi-actor environments. Here, the complementary approach consisting of multi-actor care services and digital technologies facilitates the holistic assessment of the patient’s health status and symptoms, as subjective impressions are augmented with digital measurements. As a result, the limitations of telemedicine are partially mitigated by the personal interaction of an MA with the patient, which in turn can lead to higher use intentions and adoption rates in the medical domain. Nevertheless, our findings show that a simple replacement of physical interaction between patient and MAs or GPs with digital interaction might lead to decreasing quality of care, because of a restriction of sensory information (eg, the state of a patient’s environment, odors, or mobility) and the absence of physical contact to suggest caring [73]. At worst, a strategy of digitalizing primary care only from the perspective of economical effectiveness might lead to the discrimination of vulnerable groups, such as older, chronic, and immobile patients treated through ambulant primary care.

### Telemedicine and the Key Role of MAs

The findings further indicate that the degree of responsibility and accountability with regard to actions performed by MAs when visiting a patient at home increases through the application of supportive telemedical tools. The data show that an MA, who is equipped with digital technologies such as telemedical stethoscopes or telemedical ECGs, is empowered to conduct a broad spectrum of diagnostic measures in a more autonomous and deliberate way. As a side effect, the presence of a supportive technology provides additional assurance to the MA as it augments the MA’s subjective assessments with objective data, for instance, in the form of vital parameter measurements and visualizations. Furthermore, digital ICTs are able to reassure MAs by providing an enhanced amount of information about a patient, for example, mobile apps of EHRs or an enhanced remote interaction between the GP, MA, and patient by bridging spatial and temporal limitations [75]. Another interesting implication drawn from our results is that an MA potentially possesses high degrees of both medical and technical competence. Hence, in multi-actor treatment settings involving GPs, MAs, and patients, an MA who is skillful in using, understanding, and explaining digital technologies and their purpose is able to bridge gaps in technology competence and adoption behavior that occur on both the side of the care provider (ie, GP) and the side of the consumer (ie, patient).

Considering the major challenges that arise from the individual use of telemedicine, such as the insecurity of patients (and their relatives) to assist in a remote examination by manipulating technology [76], our findings suggest that technology-mediated delegation might be a suitable way to improve workflows of primary care physicians and address issues that arise from remote consultations. In general, the literature has shown that practitioners, as well as patients, have certain reservations when it comes to forming attitudes and use intentions toward digital health care technologies. On the professional side, empirical results indicate that some GPs think that a large portion of their patients, especially older patients, are not able to operate digital technologies, which hinders the effectiveness and progress of treatments [77]. On the patient side, low technology adoption rates can occur, inter alia, because of the desire to maintain a personal and direct relationship to their GP or the lack of familiarity with the respective technologies [71]. In many cases, patients do not see the benefit that digital technologies can have on their treatment and, thus, also on their health status and progression, because of their preuser status and unfamiliarity with innovative digital health care technologies [78,79]. Therefore, the integration of factors related to technology adoption in multi-actor settings, such as external perceptions of technological competence, might provide theoretical insights that could explain variations in the explanatory power of elaborated acceptance models with regard to health care technologies [47]. In this regard, our results indicate that the approach of combining multi-actor treatment settings with supportive digital technologies can bridge structural and perceptual gaps as well as a possible digital divide. As telemedicine becomes increasingly important for primary care, older patients may face disadvantages with regard to the delivery of vital care because of the underutilization of internet use and digital technologies [74,80]. Patients are no longer obliged to adopt technologies themselves and can stick with familiar treatment patterns while maintaining direct personal contact with the treating person. Especially for older patients or patients with chronic diseases lacking competence or interest in digital technologies [74,81], technology-mediated delegation might be an essential step to adopt innovative digital approaches to primary care. In addition, GPs are more likely to integrate technological tools into their working routines, as they trust their MAs to use them efficiently. Hence, the perceived lack of willingness and the ability to adopt digitalized treatments can be resolved.

### Challenges Regarding Telemedicine Adoption

Furthermore, our results suggest that the adoption of digital technologies by GPs and MAs may be partially explained by the theoretical concept of bounded rationality. Considering the assumption that human decisions are not entirely based on a rational balancing of costs and benefits, but on heuristics and cognitive simplifications as well [82], the tendency to prefer familiar technologies, called the status quo bias, is known to have a powerful effect and could explain user resistance [83]. Most participants in our studies found the technologies that they had already learned how to integrate in their daily work routine especially relevant and, therefore, potentially assessed the subjective relevance of unfamiliar digital technologies, such as telemedical applications, from the perspective of their status quo. While anchoring their subjective perception of usefulness and relevance to familiar technologies [84], technologies containing innovative characteristics that are dissimilar to elaborated ones are possibly rejected. Thus, considering that the evaluation and adoption of technologies partially depend on heuristics in decision-making that are often applied subconsciously and are thus difficult to externalize and become aware of [85], additional barriers occur that potentially hamper the comprehensive adoption of telemedicine in multi-actor care. Therefore, to increase the attraction of innovative technologies in primary care, providers might consider the importance of the integrability of their products into the existing routines and communicative structures of their customers. The possibility of using digital technologies without an obligation to eventually buy them might hold an opportunity for customers, that is, GPs and the staff of their practices, to experimentally incorporate a new technology to overcome the status quo bias and the anchoring effects to which they are subject. On the contrary, as the aforementioned heuristics serve the goal of making fast and efficient decisions and in the light of lacking information regarding the application and performance of a given technology [86,87], it should be further taken into account that in some specific cases, digitally complementing treatments in multi-actor care do not represent a feasible and effective way of making things better, but instead introduces new forms of overhead and uncertainty.

### Theoretical and Practical Implications

Through our empirical findings, we provide insights into digital technologies and their potential for multi-actor delegation processes in primary care in relation to research, medical practice, and the development and design of technology. For theoretical reasoning, our paper points out several factors that are relevant for expanding the understanding of technology use by medical professionals. Within the context of multi-actor approaches to delegation in primary care, the results suggest that MAs facilitate the use of and access to health care technology for patients. There, MAs might even be able to compensate for a possible lack of technological competence or skills and for the resistance of patients bypassing obstacles related to usability [88]. In this regard, our results may be used to expand or contextualize theories concerning technological adoption used for telemedicine [47], such as the Technology Acceptance Model or the unified theory of acceptance and use of technology. Technology adoption and use appear to be reciprocal processes involving external perceptions of patients and their hypothetical reactions to the use of digital technologies by medical professionals, which might be considered to enrich theoretical models. In addition, we explored an anchoring effect [84] concerning the adoption of unfamiliar technologies by GPs and MAs, indicating that the adoption of digital technologies in health care might comprise cognitive biases that need to be further differentiated and operationalized to improve the predictive power of elaborated theoretical models.

From a practical point of view, our findings indicate a strong potential and benefit of using digital technologies, especially those used for telemedical examinations and video consultation for delegation processes in ambulant primary care. This holds not only for GP-MA interaction but also between GPs and nurses or registered nurses and nursing assistants. Owing to a shift in the organization of health care that can be noticed in North and Middle European, or North American countries, the relevance of team-based primary care increases [89-91]. Our study suggests that technology-mediated delegation and care processes might be a suitable way to ensure comprehensive personal care and effectively deal with the challenges of time pressure, increasing case complexity, and shortage of medical professionals. In this regard, our results are valuable for the strategic alignment of health care providers, health insurance companies, companies developing telemedicine applications, or politics. Moreover, the potential discharge of health care systems’ resources through differentiated and needs-based care has become increasingly important with respect to the ongoing COVID-19 pandemic. The ability to guarantee adequate care for patients at home through MAs utilizing telemedicine might increase time-efficient treatment and accurate monitoring of patients while decreasing inequalities concerning personal technological competence or affinity between younger and older patients. Within the multi-actor processes of primary care, digital technologies can enrich the subjective assessment of a patient by optimizing the objective measurements of medical data and by providing a more effective way to communicate and document. By comprehending the subjective interaction between MAs and patients, specific prerequisites for innovative digital technology use might change or be omitted because of the medical and technological competence of MAs. Nevertheless, our study suggests that the accuracy and reliability of the technology remain an important factor for medical professionals.

### Limitations

The results of our paper were bound to relatively small sample sizes. Although the explorative nature of our research objective matched our chosen methods of data collection and analysis, a potential bias might emerge from the fact that both studies were drawn from the same sample. In addition, data from our survey study represent only a part, but not the entire sample of our interview study, resulting in limited generalizability within the respective sample. Although GPs and MAs ensured the validity of our results from our survey study, our findings might partially reflect the tendency to confirm existing knowledge (from our interview study), rather than disprove them, known as confirmation bias [92]. Therefore, our selected method for data analysis (MDU) generally implies the risk of subjective interpretation and needs to be discussed in future research. Furthermore, as all participants in our studies were associated with a regional project on the digitalization of health care technologies for delegation processes in ambulant medical care, participants might represent opinions and perceptions that tend to be more optimistic and interested in the ongoing process of digitalization. In addition, we concentrated on a sample related to specific characteristics, that is, GPs and MAs in rural German areas. To extend the validity of our findings, a quantitative approach with a large sample size might provide insights into the generalizability of our findings and optimize the representativeness of our MDU model. As our study provides insights into the process of technology adoption through incorporation and shows the importance of practical experience for technology adoption, an interventional pre-post study might further enrich our findings.

### Conclusions

Our study explores the potential of using digital technologies in primary care delegation processes. Interviews with GPs and MAs revealed the complex situational role of technology within these delegation processes. Although the results suggest that the importance of ICT is increasing because of its ability to remove spatial and temporal limitations, telemedical solutions appear to be promising, as they enable video consultation or automated transfer of medical data. In addition, telemedical solutions have the potential to facilitate direct patient treatment by merging medical and social competence to overcome demographic and structural changes, as well as to overcome deficits in patients’ technological competence. Therefore, digital technologies assist in finding innovative, case-sensitive, and cost-effective methods of treatment in primary care. Nonetheless, our study revealed the contextual nature of technology use in primary care. The adoption and implementation of technology underlie reciprocal processes involving different attitudes and perceptions within multi-actor settings. Furthermore, the results suggest that these attitudes and perceptions might be biased because of the underlying needs for action that are unique to medical treatments. Consequently, our study provides a foundation for further investigation of relational characteristics within multi-actor settings in primary care. Finally, practical suggestions are made to improve the development and distribution of innovative technologies for medical delegation processes.

## Appendix

Multimedia Appendix 1 Interview guidelines.

## Abbreviations

ECG: electrocardiogram
EHR: electronic health record
GP: general practitioner
ICT: information and communication technology
MA: medical assistant
MDS: multidimensional scaling
MDU: multidimensional unfolding
