# Supplementary material for: Investigating the Use of Telemedicine for Digitally Mediated Delegation in Team-Based Primary Care: Mixed Methods Study
Source: J Med Internet Res. 2021 Aug 26;23(8):e28151. doi: 10.2196/28151 (PMC8430853; doi:10.2196/28151)
Supplement: Multimedia Appendix 1 [file jmir_v23i8e28151_app1.docx]

**Multimedia Appendix 1: Interview guidelines.**

**1. Personal questions**

- How old are you?
- How long have you been in your profession and how long have you been working in your current position / for your current employer?
- Which activities in your job are particularly important to you / which do you consider essential?
- What role does digitalization play in your work?

**2. Processes of medical care and delegation**

- Can you describe a typical working day from your / the MA’s perspective?
- Can you describe a home visit to a patient?
- How do the patients you visit at home differ?
- Which tasks do you delegate to MAs / are delegated to you?
- In your opinion, which tasks would be suitable to be delegated in the future?

**3. Specifications/Environment/Structures**

- What has changed for your everyday work with the concept of MAs for home visits / when you became an MA that is allowed to make home visits?
- What has changed for patient care with the concept of MAs for home visits / when you became an MA that is allowed to make home visits?

**4. Technologies and their impact on professional’s self-image**

- What experience do you have with the use of technologies in everyday work?
- How did you come into contact with the principle of telemedicine?
- What is your opinion on telemedicine?
- What are benefits/potentials/risks of telemedicine?
- What relevance does telemedicine have for your everyday work?
- When you briefly review your time in practice. How have technologies changed your everyday work?
- What would technology have to do to help you in your everyday work? What would you like to use?
- What are important conditions for a successful and sustainable use of telemedicine from your perspective / from the perspective of patients?

**5. Patient-GP/Patient-MA relationship and interaction**

- How would you describe the relationship with patients?
- In your opinion, what role can technology play in your relationship with patients?
- How do patients perceive the technology they are using? / How do patients react to technology that you use?
- How would you describe differentiating, individualizing, and generalizing effects of technology with regard to primary care?

**6. Open questions**

- To what extent do you think digital technologies will change the professional profile of doctors and MAs?
- What do you wish for regarding the digitization of health care in general?
- How do you envision future primary care?
- How do you imagine your own everyday work in the future?
